# Swallowed dental prosthesis causing late oesophageal obstruction and tracheo-oesophageal fistula in a young patient

**DOI:** 10.1093/jscr/rjag946

**Published:** 2026-10-19

**Authors:** Harish Prasanna, Daniel Boyle, David Bowrey

**Affiliations:** Leicester Medical School, University of Leicester, George Davies Centre, Lancaster Road, Leicester LE1 7HA, United Kingdom; Leicester Medical School, University of Leicester, George Davies Centre, Lancaster Road, Leicester LE1 7HA, United Kingdom; Leicester Royal Infirmary, University Hospitals of Leicester NHS Trust, Infirmary Square, Leicester LE1 5WW, United Kingdom

**Keywords:** oesophageal foreign body, dental prosthesis, denture ingestion, tracheo-oesophageal fistula, oesophagectomy, delayed diagnosis

## Abstract

A 25-year-old man presented to the emergency department after a rugby accident having swallowed a dental plate. Initial assessment at the time of ingestion, including a chest radiograph, was unremarkable and the plate was presumed to have been expectorated. Five years later he presented with progressive dysphagia, recurrent food bolus obstruction, and haemoptysis. Computed tomography of the thorax performed 5 years later revealed circumferential oesophageal thickening and a large inflammatory mass around a retained radio-opaque foreign body at the level of the carina, with an associated tracheo-oesophageal fistula. Endoscopy confirmed near-complete obstruction of the oesophagus. He underwent a right thoracotomy with oesophagectomy, removal of the retained dental prosthesis and repair of the fistula. He made a slow but steady recovery and has required anastomotic dilatation for a stricture. This case highlights the risk of complications from retained oesophageal foreign bodies and the limitations of plain radiography in detecting non-radio-opaque dental plates.

## Introduction

Ingested foreign bodies, including dental prostheses, are a well-recognized cause of oesophageal obstruction [1]. They are usually recognized and managed at the time of the incident, or shortly after, because they present with acute symptoms such as dysphagia or chest discomfort [2]. Prolonged retention within the oesophagus is rare but may lead to progressive complications [3]. We report a retained dental prosthesis causing late oesophageal obstruction and fistulation 5 years after ingestion, illustrating the importance of maintaining clinical suspicion and using appropriate imaging when initial investigations are inconclusive.

## Case report

A 25-year-old man accidentally swallowed a dental prosthesis while playing rugby. He attended the emergency department, was assessed and was discharged following a normal chest radiograph; the prosthesis was presumed to have been expelled as it was not visible on imaging.

Over the following years he had intermittent dysphagia, which he attributed to the original event, and did not seek further medical attention. Five years later his symptoms progressed, with several episodes of food bolus obstruction, a persistent cough and haemoptysis. His general health and his weight were unchanged. The patient had a normal radiograph when he represented as well (Fig. 1).

**Figure 1:**
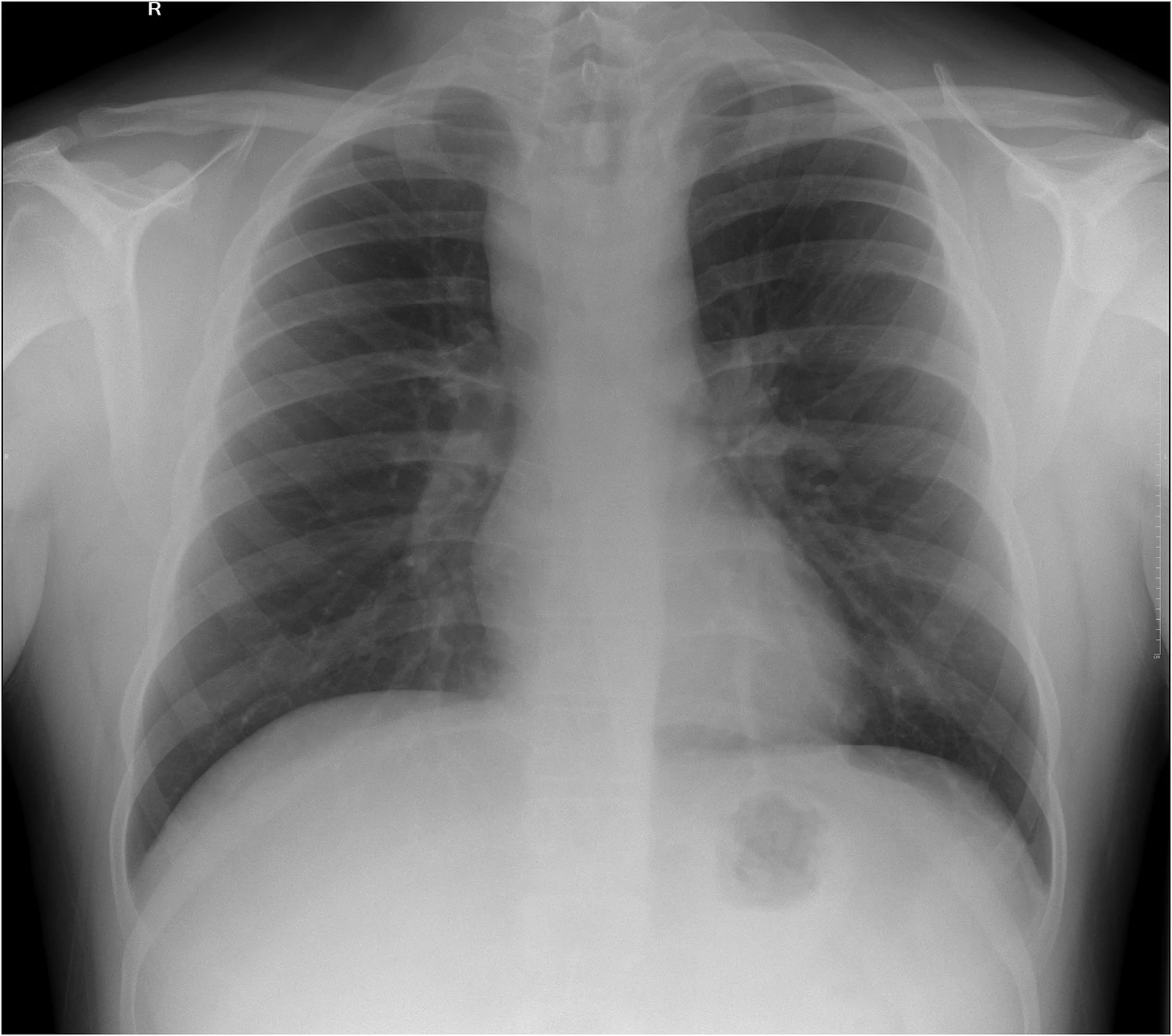
Postero-anterior chest radiograph. The lung fields, mediastinal contours, and diaphragm are unremarkable, and no radio-opaque foreign body is demonstrated, illustrating the failure of plain radiography to identify the retained dental prosthesis.

Computed tomography (CT) of the thorax, performed 5 years after the ingestion to investigate haemoptysis, demonstrated circumferential thickening of the proximal and mid-oesophagus, measuring 4.53 cm in transverse diameter. The same CT examination also demonstrated an inflammatory mass at the level of the carina surrounding a radio-opaque foreign body, with an associated tracheo-oesophageal fistula (Fig. 2 and 3). Endoscopy identified a near-complete occlusion of the oesophageal lumen from the upper to the mid-oesophagus (Fig. 4).

**Figure 2:**
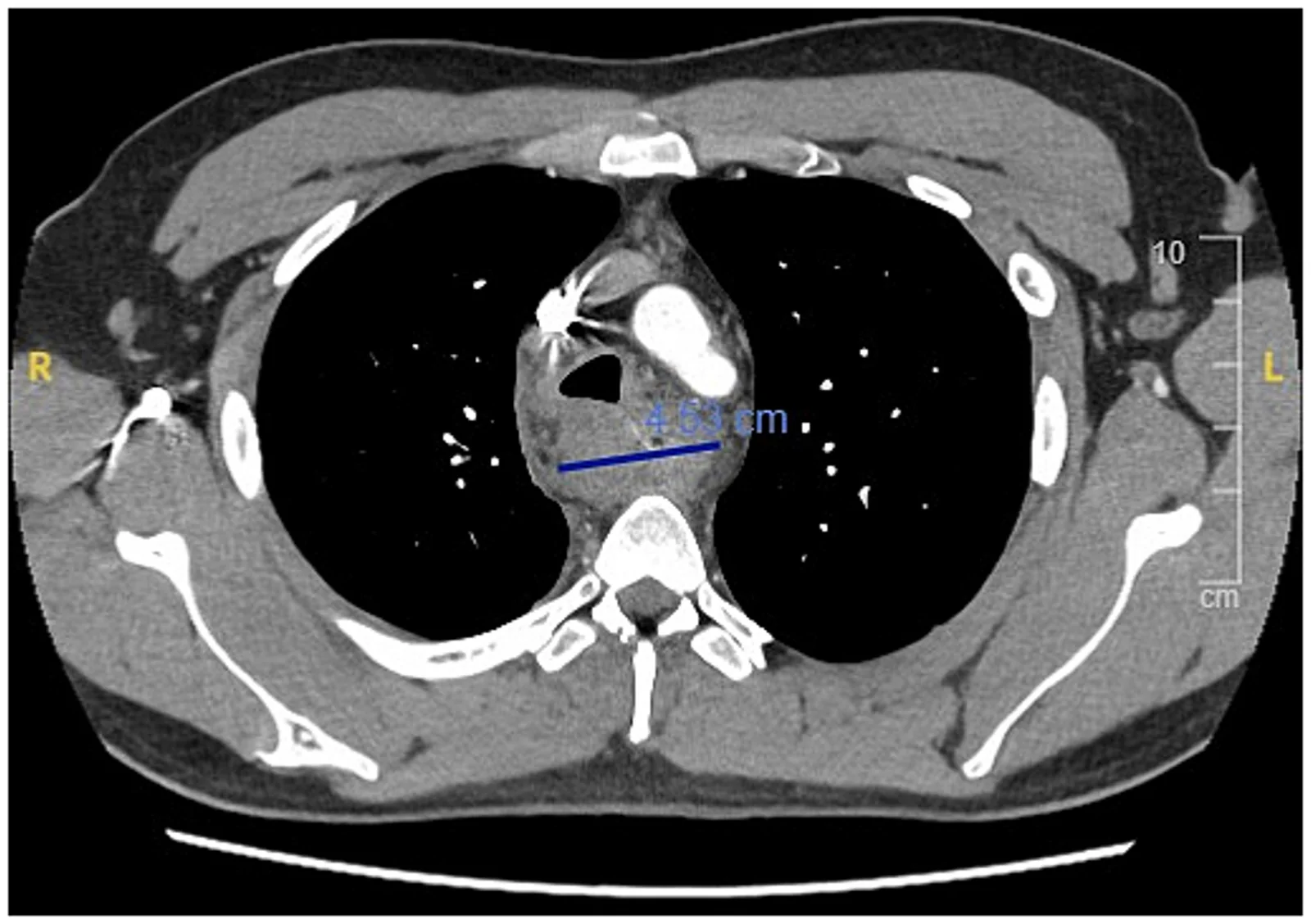
Axial contrast-enhanced CT of the thorax at the level of the upper mediastinum, demonstrating circumferential soft-tissue thickening of the proximal oesophagus posterior to the trachea. The calliper indicates the transverse diameter of the thickened segment, 4.53 cm.

**Figure 3:**
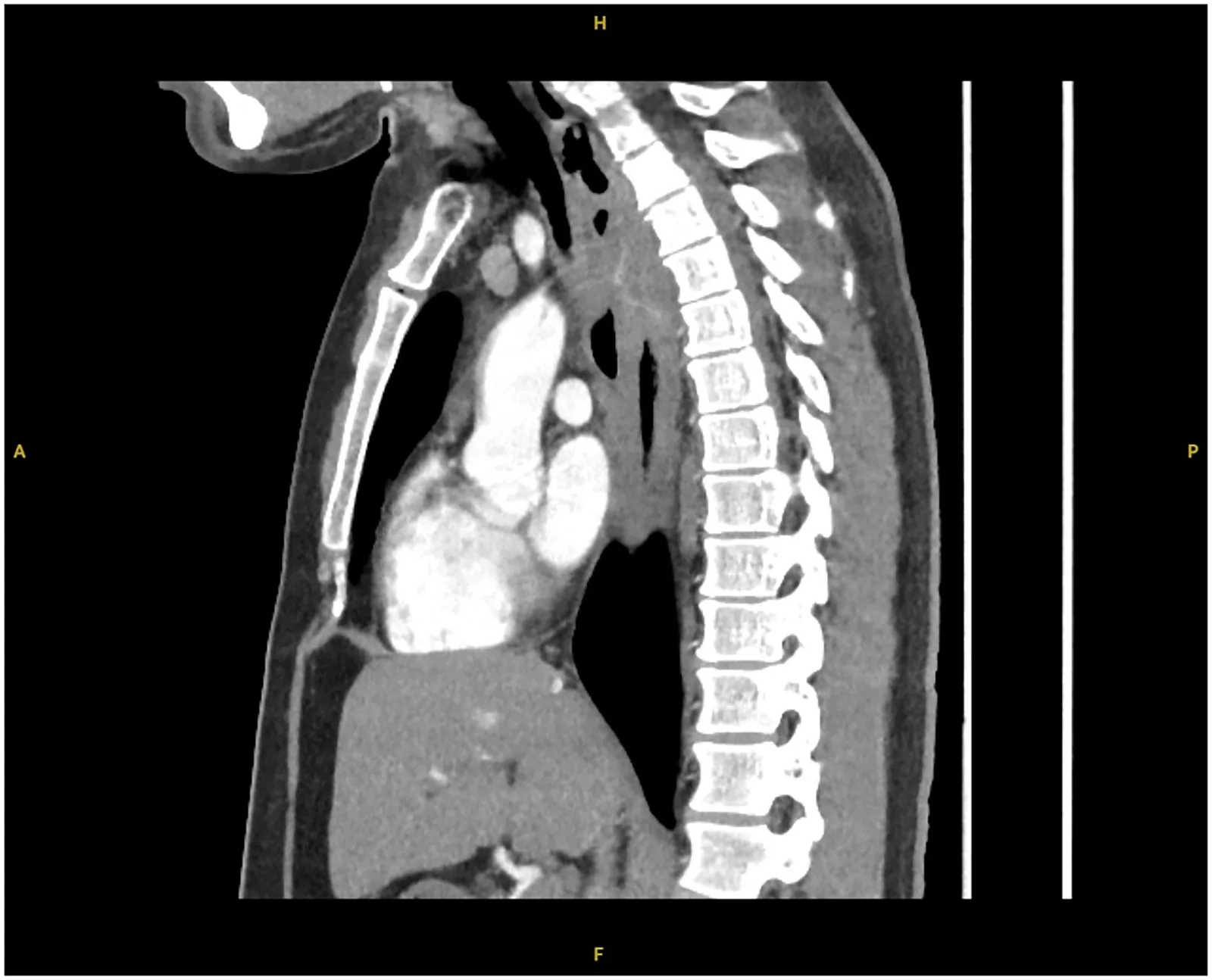
Sagittal contrast-enhanced CT of the thorax, demonstrating the retained radio-opaque dental prosthesis at the level of the carina, with surrounding inflammatory soft-tissue change and circumferential thickening of the adjacent oesophagus.

**Figure 4:**
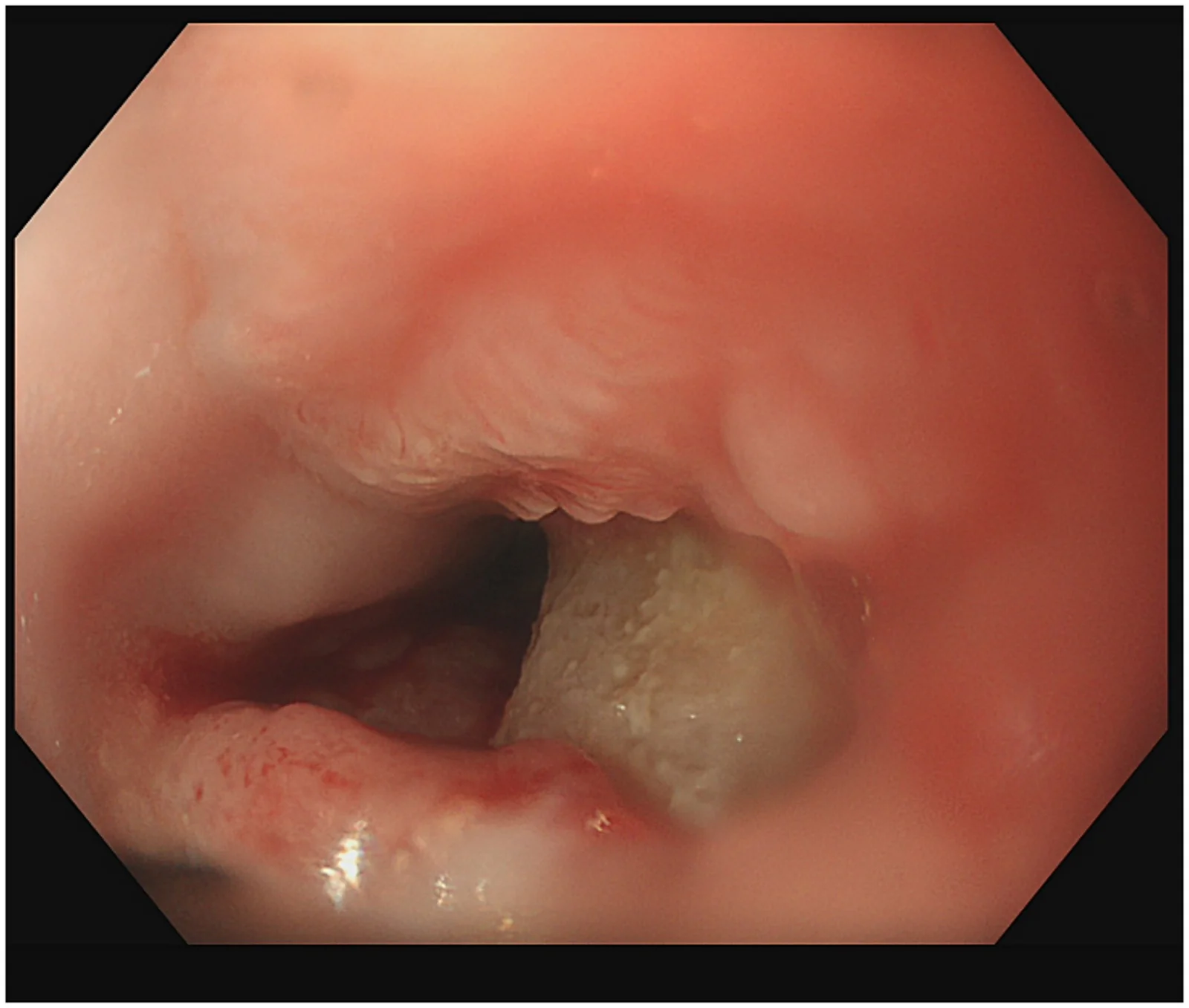
Endoscopic view of the oesophagus demonstrating near-complete occlusion of the lumen by an inflammatory mass, with adjacent mucosal erythema.

The principal differential diagnosis was oesophageal malignancy: progressive dysphagia, luminal narrowing, and nodular mucosal change raised concern for carcinoma [4]. Biopsies showed only chronic inflammatory change. Eosinophilic oesophagitis was considered given his age and symptoms, but the endoscopic findings were not characteristic of that condition [5]. CT excluded external compression by a mediastinal mass or lymphadenopathy. Re-evaluation of the history alongside the imaging findings established the diagnosis of a chronically retained oesophageal foreign body: the prosthesis ingested 5 years earlier had never been expelled. He had no other medical history.

The case was discussed at the upper gastrointestinal multidisciplinary team meeting and definitive surgical management was planned (Fig. 5). He underwent a right thoracotomy with oesophagectomy, removal of the retained prosthesis and repair of the fistula [3]. A photograph of the removed dental prosthesis following extraction is shown in (Fig. 6).

**Figure 5:**
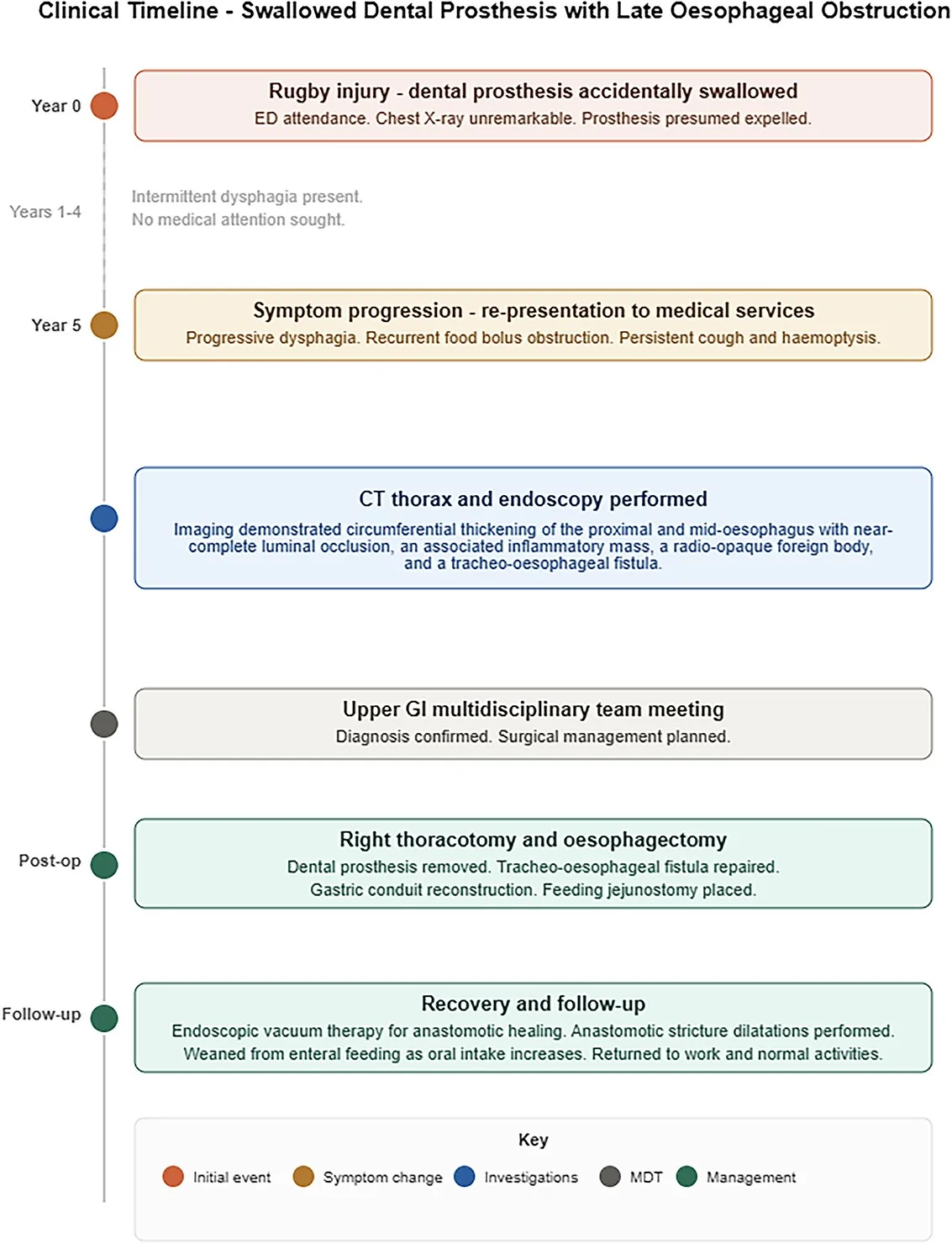
Clinical timeline illustrating the 5-year course from ingestion of the dental prosthesis to surgical management and recovery.

**Figure 6:**
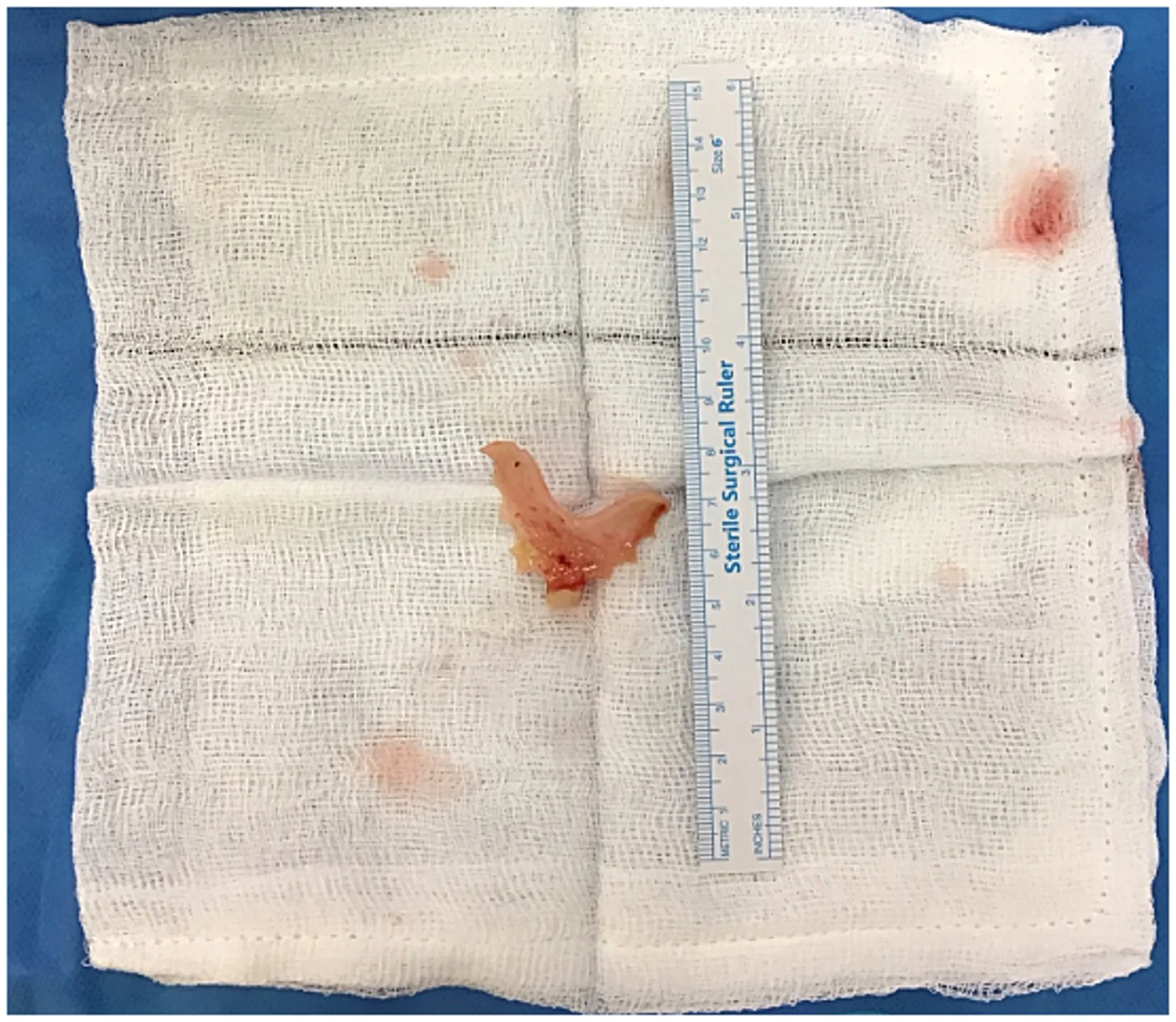
Removed dental prosthesis following surgical extraction, shown on surgical gauze alongside a ruler for scale.

The oesophagus could not be salvaged because of extensive fibrosis, and reconstruction was with a gastric conduit. A feeding jejunostomy was placed to provide enteral nutritional support, and a prophylactic endoscopic vacuum sponge was sited at the level of the anastomosis to control any leak and promote healing.

Endoscopic vacuum therapy was required for several weeks [6]. He was discharged home on a programme of jejunostomy feeding and has required several anastomotic stricture dilatations. At latest review he is being weaned off enteral feeding as oral intake increases, his weight is stable, and he has returned to his occupation and normal activities.

## Discussion

Foreign body ingestion is a common presentation, particularly in the emergency department, and most ingested objects pass spontaneously or are identified early and managed acutely [7]. Oesophageal impaction represents the clinically significant subgroup because of the risk of obstruction and perforation [8]. Dental prostheses carry a higher risk owing to their irregular shape and rigid structure, which makes impaction and local trauma more likely [1, 9].

Symptoms usually develop immediately or soon after ingestion, prompting early investigation and intervention [7]. Delayed presentations are uncommon and typically occur over hours to days, with occasional reports extending to weeks or months. Presentation beyond 1 year is exceptionally rare, and retention undetected for several years is very unusual [10, 11].

Delayed complications arise from chronic mechanical irritation and pressure necrosis caused by the impacted object. Over time this produces mucosal ulceration, local inflammation and progressive fibrosis, with luminal narrowing and dysphagia [12]. In more severe cases, the object erodes into adjacent structures and a fistula forms [8, 13]. The tracheo-oesophageal fistula seen in this patient indicates advanced local disease and carries substantial morbidity [3].

The delayed diagnosis in this patient followed reliance on a single chest radiograph. Dental prostheses are frequently radiolucent or only partially radio-opaque [1, 9], which can lead to false reassurance and discharge when clinical suspicion is not maintained [3]. Current evidence supports early endoscopic evaluation or CT where the history suggests foreign body ingestion, even when initial imaging is negative [2, 7].

Published experience of long-term retained oesophageal foreign bodies causing significant complications is limited [14]. Most reported delays are of days to a few months, and only a handful exceed 1 year. These include a ring-pull from a beverage can retained for 26 months, an object usually visible on plain radiograph [15], and a partially degraded foreign body measuring 25 cm that caused progressive dysphagia without postoperative complication [11]. Only one previous report describes a comparably delayed presentation of denture impaction [3].

Management of retained oesophageal foreign bodies depends on the duration of impaction, the object itself, and the presenting complications. Endoscopic retrieval is preferred for acute cases, but chronic impaction with fibrosis, obstruction or fistula formation often requires surgical intervention [2, 7]. In this case, the extent of oesophageal involvement and the presence of a tracheo-oesophageal fistula required a combined thoracic and abdominal approach [3].

This case adds to the limited literature on chronically retained oesophageal foreign bodies by documenting a retention period of 5 years resulting in severe local complications. It carries two clinical messages. First, a normal plain radiograph does not exclude foreign body retention, particularly for radiolucent objects. Second, persistent or progressive dysphagia following a suspected ingestion warrants thorough re-evaluation. Greater clinical awareness and a lower threshold for CT or endoscopy at initial presentation might have prevented 5 years of retention and the loss of the oesophagus.
